# Diabetes‐related barriers to cancer screening in women with type 2 diabetes: A qualitative interview study

**DOI:** 10.1111/bjhp.70088

**Published:** 2026-06-10

**Authors:** Rebecca Spencer, Georgina Jones, Siobhán McHugh, Ramzi A. Ajjan, Rebecca J. Birch, Laura Ashley

**Affiliations:** ^1^ School of Humanities and Social Sciences Leeds Beckett University Leeds UK; ^2^ Leeds Institute of Cardiovascular and Metabolic Medicine, School of Medicine University of Leeds Leeds UK; ^3^ Leeds Institute for Medical Research, School of Medicine University of Leeds Leeds UK

**Keywords:** bowel screening, breast screening, cancer, cancer screening, cervical screening, diabetes, qualitative

## Abstract

**Objectives:**

People with type 2 diabetes (T2DM) are more likely to develop breast and bowel cancers. Despite this, cancer screening participation is lower among women with diabetes than among women without diabetes, indicating diabetes‐related barriers to screening, but little research has examined this. This study aimed to identify and understand diabetes‐related barriers to cancer screening, and potential ways to address these, among women with T2DM.

**Design:**

In‐depth qualitative interviews.

**Methods:**

Semi‐structured interviews with 25 women with T2DM, aged 50 to 74 years, living in England. Participants were recruited via diabetic eye screening clinics and community advertisement. Data were analysed to develop themes, using the framework method.

**Results:**

Women with T2DM were often living with an accumulated high burden of illness and its treatment, due to diabetes and comorbidities, which reduced their capacity to participate in cancer screening (e.g. physical and psychological capacities; practical resources). Having diabetes could complicate taking part in screening tests for some people (e.g. physical difficulties during screening related to diabetes, its treatment, complications or comorbidities; having to consider glycaemic control during appointments). There appeared to be underappreciation of the T2DM‐increased risk of cancer, and limited cancer screening promotion within diabetes care.

**Conclusions:**

Despite self‐reported cancer screening uptake being high among study participants, having diabetes appeared to heighten common barriers to cancer screening (e.g. travel‐related, logistical and scheduling barriers), whilst also posing additional unique barriers (e.g. diabetes‐related stigma and embarrassment). Several potential strategies are suggested to improve cancer screening informed decision‐making and participation among people with T2DM.

**Statement of Contribution**

**What is already known on this subject?**

- People with type 2 diabetes (T2DM) are more likely to develop breast and bowel cancers and experience worse cancer‐related outcomes.
- Women with diabetes are less likely to participate in routine cervical, breast and bowel cancer screening.
- Little qualitative research has explored diabetes‐related barriers to cancer screening.

**What does this study add?**

- This is the first qualitative study to examine experiences and barriers around cancer screening among women with T2DM in England.
- Having diabetes can heighten commonly experienced barriers to cancer screening and pose additional unique barriers.
- There is limited promotion of T2DM‐increased cancer risk and cancer screening within diabetes care.

## INTRODUCTION

The global incidence of cancer is growing rapidly (Sung et al., 2021), and in the UK, one in two people will now develop cancer in their lifetime (Ahmad et al., 2015). Cancer‐related morbidity and mortality can be reduced by routinely screening asymptomatic individuals (Loud & Murphy, 2017). Cancer screening aims to prevent the development of cancer, by finding pre‐cancerous abnormalities, or detect it at an earlier stage, when it is generally more treatable and survivable (Wender et al., 2019). Many countries provide screening for a range of cancer types (e.g. breast, cervical and colorectal cancers), via organized programmes (e.g. the UK) or opportunistically (e.g. the USA) (Miles et al., 2004). Despite the benefits of cancer screening, a sizeable proportion of eligible individuals do not participate, with numerous practical, social, emotional and psychological barriers identified among the general population (Young et al., 2018). In 2022 to 2023, coverage of cervical, breast and bowel cancer screening in England was approximately 69% (NHS Digital, 2023), 67% and 72% (Office for Health Improvement and Disparities, 2024), respectively. Disparities in cancer screening uptake (Young & Robb, 2021) include lower participation rates among people with diabetes (Bhatia et al., 2021; Parrent et al., 2025).

Diabetes mellitus is a chronic metabolic condition which requires high levels of self‐management and care to maintain adequate glycaemic control to prevent or mitigate acute diabetic symptoms and long‐term health complications (American Diabetes Association, 2013; Kalyani et al., 2025). Suboptimal glycaemic control can cause unpleasant symptoms (e.g. fatigue; inability to concentrate; increased thirst) (Kalyani et al., 2025; Nakhleh & Shehadeh, 2021) which may reduce health‐related quality of life (Cannon et al., 2018). Poorly controlled diabetes also increases the risk of chronic vascular complications such as heart disease, foot ulcers that can lead to amputations, and retinopathy which may result in visual impairment or blindness (American Diabetes Association, 2013). Diabetes self‐management and care often includes monitoring blood sugar levels (e.g. via continuous glucose monitoring devices or finger‐prick tests); administration of medications (e.g. insulin; metformin), which can have side effects (e.g. nausea; diarrhoea); dietary adjustments; and attending health screening and review appointments (e.g. diabetic eye screening; foot checks) (American Diabetes Association, 2014; Kalyani et al., 2025). Global prevalence of diabetes is high and rising (Saeedi et al., 2019). In England, an estimated 3.8 million people over age 16 have diabetes; this figure is projected to increase to 4.9 million by 2035 (Public Health England, 2016), with 90% having type 2 diabetes mellitus (T2DM) (Chatterjee et al., 2017). People with T2DM have an increased risk of developing cancer compared to those without diabetes, particularly colorectal (Larsson et al., 2005) and breast (Larsson et al., 2007) cancers. Although this increased risk may be partly due to shared risk factors between T2DM and cancer (e.g. obesity; smoking; increasing age), research is ongoing into direct biological links between the two conditions (e.g. hyperglycaemia; hyperinsulinemia; inflammation; sex hormone dysregulation) (Suh & Kim, 2019; Wang et al., 2020). Furthermore, comorbid diabetes is associated with worse cancer outcomes (Shahid et al., 2021; Vissers et al., 2016), including increased mortality (Harborg et al., 2024).

Despite the T2DM‐increased risk of bowel and breast cancers, many people with diabetes do not participate in cancer screening as recommended (Adjaye‐Gbewonyo et al., 2013; Miller & Pinsky, 2022). Studies suggest that, compared to those without diabetes, people with diabetes are less likely to have ever participated in, or to be up to date with, cancer screening (Chuck et al., 2017; Jiménez‐Garcia et al., 2009; McDaniel et al., 2021). For example, in an analysis of data from the English Longitudinal Study of Ageing, people with T2DM had significantly lower rates of ever participating in (63% vs. 76%), or being up to date with (60% vs. 72%), colorectal cancer screening than people without diabetes (von Wagner et al., 2020). A recent systematic review and meta‐analysis by Bhatia et al. (2020) concluded that women with diabetes were less likely to receive recommended cervical, breast and colorectal cancer screening, compared to women without diabetes, particularly in countries where screening is conducted via organized programmes. However, colorectal cancer screening rates among men did not significantly differ between those with and without diabetes; the reasons for this gender difference are unclear.

Lower cancer screening rates among people with diabetes persist after controlling for other factors known to influence screening uptake (e.g. socioeconomic status (Chan et al., 2014); comorbidities; body mass index), suggesting there are diabetes‐related barriers to cancer screening (von Wagner et al., 2020). Very little qualitative research has sought to elucidate such barriers. Although studies examining views on cancer screening have included people with diabetes alongside participants with other health conditions (Armin et al., 2014; Kumar & Mohammadnezhad, 2022; Shaw et al., 2012), to our knowledge, only two USA‐based studies have focused exclusively on people with diabetes. Marshall (2014) conducted focus groups with women with diabetes to understand perceived determinants of cervical and breast cancer screening, whilst Mkuu et al. (2024) assessed the acceptability of human papillomavirus (HPV) self‐collection among Black women with T2DM and social vulnerability. Despite providing useful initial insights into factors which may influence screening participation among people with diabetes (e.g. perceived importance of screening; provider encouragement; perceived personal cancer risk), Marshall's (2014) study did not specify participants' diabetes type nor explore bowel screening. Furthermore, findings from these studies may not be fully applicable to countries offering organized cancer screening programmes and/or free, universal healthcare, such as the UK (Priaulx et al., 2020). This has been identified as a knowledge gap, with von Wagner et al. (2020) recently calling for qualitative research to elucidate why people with diabetes receive less cancer screening. Especially given the T2DM‐increased risk of cancer, understanding and addressing lower cancer screening uptake among people with diabetes is imperative (Parrent et al., 2025). We sought to address this gap by conducting in‐depth qualitative interviews with women with T2DM in England, to identify and understand diabetes‐related barriers to cervical, breast and bowel cancer screening, and potential ways to address these.

## MATERIALS AND METHODS

### Participants, sampling and recruitment

Participants were women aged 50–74 years, with self‐reported T2DM, living in England and therefore eligible to receive at least one type of routine NHS cancer screening (regardless of screening participation history). The current study focused exclusively on women due to findings from the recent systematic review and meta‐analysis by Bhatia et al. (2020) suggesting that women with diabetes, but not men with diabetes, are less likely to participate in cancer screening, and pragmatic reasons (e.g. men are only eligible to receive bowel screening, so would require a different interview topic guide). Participants needed to have capacity to give informed consent and speak English well enough to participate in an interview. Consistent with similar previous studies (e.g. Bennett et al., 2023; O'Donnell et al., 2023), we aimed to recruit up to 25 participants, to enable some sample diversity on key participant characteristics (e.g. age, cancer screening participation history), and likely sufficient data saturation to address the research questions (Hennink & Kaiser, 2022). To facilitate the recruitment of a diverse sample of participants, the study was advertised in diabetic eye screening clinics in eight NHS Trusts in England (as a poster and/or pile of flyers), on social media (e.g. Facebook; X/Twitter) and via diabetes charities (e.g. Diabetes UK Support Forum). Ethical approval was obtained from the North East – Tyne and Wear South NHS Research Ethics Committee (ref: 22/NE/0037) on 16/03/2022.

### Design and data collection

The study employed an in‐depth qualitative interview design. Each participant took part in one semi‐structured, audio‐recorded interview to explore their perspectives and experiences around cancer screening. We examined cervical, breast and bowel cancer screening, as has been done in previous interview studies examining barriers to cancer screening participation (e.g. Clifton et al., 2016; Kirkegaard et al., 2023; Kotzur et al., 2020). Interviews focused on identifying and understanding diabetes‐related barriers to cancer screening, and potential ways to address these, and were informed by a topic guide, used flexibly to capitalize on the natural conversation flow. To facilitate a thorough exploration of barriers and enablers to cancer screening, the topic guide was informed by previous literature (e.g. Honein‐AbouHaidar et al., 2016; Sarma, 2015; Spencer et al., 2024) and drew upon the Theoretical Domains Framework (TDF) (Atkins et al., 2017; Cane et al., 2012; Michie et al., 2005). As recommended by McGowan et al. (2020), interviews used broader, open‐ended questions related to the TDF domains, rather than highly structured questions systematically addressing each domain in turn, in relation to each cancer screening type. This approach avoided the interviews becoming too long or repetitive, and overlooking important factors not covered by the TDF. The use of individual interviews enabled in‐depth coverage of the full topic guide for each participant, which would not have been possible using alternative qualitative data collection methods, such as focus groups. Furthermore, individual interviews provide participants with an accessible and supportive environment, which may encourage more open discussion of sensitive topics (Kruger et al., 2019).

Initially, interviews briefly explored participants' experiences of living with diabetes and of being invited to cervical, breast and/or bowel cancer screening. To enable description of the study sample, participants were also asked to self‐report their diabetes characteristics, sociodemographic characteristics that have previously been shown to influence cancer screening participation (i.e. age; ethnicity; relationship status; employment status; highest level of education; the presence of comorbidities) (Helgestad et al., 2024; Young & Robb, 2021), and their cancer screening participation history. Interviews covered cancer screening decision‐making (e.g. ease or difficulty of deciding whether to participate in cancer screening), including perceived importance of cancer screening and perceived personal risk of developing cancer, particularly as someone with T2DM. Participants were asked about their experiences of barriers and facilitators to cancer screening; discussion focused on elucidating diabetes‐related barriers, and included seeking participants' thoughts on potential barriers and facilitators suggested by research or other interviewees (e.g. competing priorities; reticence to undress in front of healthcare professionals; loose bowels/constipation; cancer screening information provided by healthcare professionals; family support). Participants were asked about their suggestions for potential ways to address barriers to cancer screening for women with diabetes (e.g. what changes could be made and how could these best be done?). At interview close, participants were encouraged to raise anything else they wished to say or discuss.

### Procedure

After viewing the study advertisement, interested individuals contacted the researcher directly (via email or telephone) and were sent a participant information sheet and consent form, and given the opportunity to ask questions. Participants chose the time and mode of their interview (i.e. by telephone, video call or in person). Participants gave informed consent and were made aware that they could take a break during the interview; choose not to answer any questions; end the interview at any point, without providing an explanation; and request to withdraw their data within 2 weeks of leaving the study. Participants received a £25 shopping voucher to thank them for their time and contribution.

### Analysis

Data collection and analysis were conducted concurrently, to allow the analysis to inform subsequent data collection. Framework analysis (Ritchie & Spencer, 1994), a form of thematic analysis widely utilized in applied health research, was conducted following the seven stages described by Gale et al. (2013): (1) verbatim transcription of the interviews; (2) transcript familiarization; (3) coding; (4) developing a working analytical framework; (5) applying the analytical framework; (6) data charting into a framework matrix; and (7) data interpretation.

Inductive codes were generated by R.S. and L.A., who each independently read a unique set of five transcripts and subsequently compared and discussed their initial thoughts on potential codes. Some deductive codes were also developed, based on the topic guide and existing literature. As recommended by McGowan et al. (2020), TDF domains were not included as a priori codes to avoid factors not covered by the TDF being disregarded. Codes were finalized iteratively by R.S. and grouped into categories to develop a working analytical framework, which was reviewed, discussed and agreed upon by R.S., L.A. and G.J., and subsequently applied to all transcripts by R.S. Data were charted into a framework matrix, with data relating to each code/category summarized by participant. S.M. reviewed the coding and charting of three transcripts for consistency and thoroughness. The research team developed themes and subthemes inductively from the data, supported by illustrative quotes. The use of the framework matrix ensured thorough engagement with all of the data for all participants and facilitated comparisons to be made both between and within participants and codes/categories. NVivo‐12 and Microsoft Excel were used to manage data.

### Reflexivity

All interviews were undertaken by R.S., a female Psychology PhD student from a non‐clinical background, who did not have a prior relationship with any participant. R.S. did not have personal experience of living with T2DM or participating in cancer screening; due to being naïve to participants' experiences in these ways, R.S. may have asked more questions and participants may have taken on an ‘educator’ role (Fisher et al., 2022). However, R.S. does have personal experience of having cancer and accessing health care, which, although unrevealed to participants, may have facilitated rapport through interviewer increased empathy and shared understanding about experiencing illness and undertaking illness‐related work. As R.S. was not a clinician, participants may have felt able to talk more openly about the healthcare system or healthcare professionals. R.S. recorded personal reflections after each interview (e.g. perceived rapport; the occasional presence of other people alongside participants) (Olmos‐Vega et al., 2023), and regularly discussed data collection and analysis with other research team members.

## RESULTS

### Participants and data collection

Twenty‐five women with T2DM were interviewed between April 2022 and January 2024. Participant characteristics are summarized in Table 1. We note that self‐reported cancer screening participation was relatively high among study participants. No participants dropped out of the study or asked to withdraw their data. All interviews were conducted by telephone (*n* = 14) or online (*n* = 11), and lasted 18–84 min (median = 55 min). No participants chose to take part in an in‐person interview.

**TABLE 1:** Participant self‐reported sociodemographic and diabetes characteristics, and cancer screening participation history (*N* = 25).

Characteristics	*N* (%)
**Age (years)**	
50–54	5 (20)
55–59	7 (28)
60–64	4 (16)
65–69	6 (24)
70–74	3 (12)
Mean age (range)	60.8 (50–73)
**Ethnicity**	
White	22 (88)
Not reported^a^	3 (12)
**Relationship status**	
In a relationship	16 (64)
Not in a relationship^b^	9 (36)
**Highest level of education**	
School/further education qualifications	12 (48)
University degree level qualifications	12 (48)
Not reported^c^	1 (4)
**Employment status**	
Not working/retired	15 (60)
Currently working^d^	10 (40)
**Number of comorbidities**	
None	2 (8)
One	6 (24)
Two	5 (20)
Three or more	12 (48)
**Approximate number of years living with type 2 diabetes (T2DM)** ^e^	
≤ 1	5 (20)
> 1 and ≤ 5	10 (40)
> 5 and ≤ 10	5 (20)
> 10	5 (20)
**Type(s) of diabetes management**	
Lifestyle changes (e.g. diet or exercise)	24 (96)
Glucose‐lowering medication (current or previous use)^f^	20 (80)
Insulin therapy	5 (20)
**Previous cancer diagnosis disclosed**	
Breast cancer	2 (8)
**Previously participated in cervical screening when invited**	
Always	20 (80)
Sometimes	4 (16)
Never	1 (4)
**Previously participated in breast screening when invited**	
Always	21 (84)
Sometimes	1 (4)
Never	1 (4)
Not previously invited	2 (8)
**Previously participated in bowel screening when invited**	
Always	13 (52)
Sometimes	2 (8)
Not previously invited	10 (40)

^a^ Three participants did not specify their ethnicity, but provided their nationality instead (i.e. British and/or Scottish).

^b^ This included participants who were single, divorced or widowed.

^c^ One participant was not asked about their highest level of education.

^d^ This included participants who were employed (full‐ or part‐time), self‐employed or semi‐retired.

^e^ The length of time that participants had been living with T2DM ranged from 1 month to 17 years.

^f^ Three participants were able to stop taking glucose‐lowering medication after making lifestyle changes.

### Analytical themes

**FIGURE 1:**
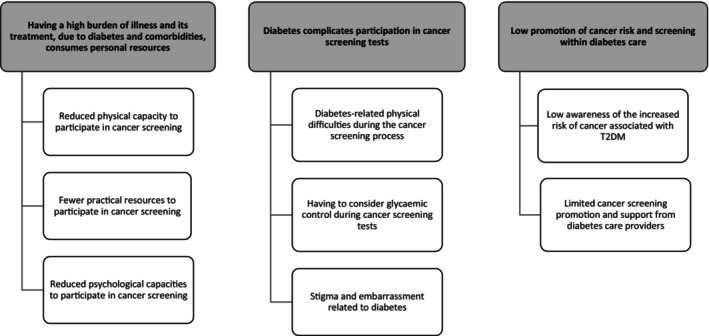
Key analytical themes and subthemes developed around diabetes‐related barriers to cancer screening participation.

The three developed themes, and their subthemes, are summarized in Figure 1, and detailed below with illustrative quotes.

### Having a high burden of illness and its treatment, due to diabetes and comorbidities, consumes personal resources

#### Reduced physical capacity to participate in cancer screening

Having a high burden of illness and its treatment, due to diabetes and comorbidities (e.g. arthritis; hypertension; chronic pain), sometimes reduced peoples' physical capacity to participate in cancer screening. Almost all women with T2DM described instances of feeling unwell or fatigued, and many experienced physical disability, which could make attending and completing screening more physically demanding. Having reduced physical capacity sometimes heightened common practical barriers to cancer screening (e.g. travel‐related, logistical and scheduling barriers). For example, feeling unwell or experiencing physical disability could make it more difficult for participants to leave their home, travel to appointments (e.g. due to challenges using public transport or being unable to drive) or physically access screening facilities (e.g. due to difficulties walking or using stairs). To buffer their reduced physical capacity, some women needed to coordinate practical support from family members, or hospital transport, to attend screening appointments. Whilst some women indicated that they would participate in cancer screening despite feeling unwell, others suggested that they may occasionally need to cancel and rearrange appointments.

Especially knowing what I feel like now with my blood [sugar levels] spiking […] It's difficult because I've got the ME [myalgic encephalomyelitis] as well, but […] if I needed to get a bus, would I have the energy to walk to the bus stop? The answer now is, not really. P2 (aged 53; living with T2DM for 1–5 years; cervical and breast screening = always; bowel screening = not previously invited)

Well, that would be difficult for me [to travel to cancer screening appointments] if I didn't have my hubby to drive me. […] Because although […] I can legally drive […] I'm far too nervous to do so, because I simply can't see anything out of this [eye]. P18 (aged 70; living with T2DM for 1–5 years; cervical, breast and bowel screening = always)

There have been times, um, that I have cancelled [appointments], um, because I am lying in bed at the time. Um, because I, I actually can't get up, I can't move without being in so much pain that I wanna cry. P28 (aged 50; living with T2DM for 1–5 years; cervical screening = always; breast and bowel screening = not previously invited)

#### Fewer practical resources to participate in cancer screening

Managing diabetes and comorbidities (e.g. attending hospital appointments, managing medications) was often time‐consuming and could also negatively impact finances (e.g. participants were occasionally unable to work due to ill health). Having fewer practical resources appeared to exacerbate common practical barriers to cancer screening (e.g. travel‐related, logistical and scheduling barriers). For example, having to attend frequent medical appointments sometimes resulted in clashes with cancer screening appointments, difficulties taking additional time off work and accumulating travel costs. Consequently, participants occasionally suggested that they may need to cancel and rearrange screening appointments or prioritize attending certain appointments over others. To mitigate the impact of having fewer practical resources, women with T2DM proposed various adaptations to cancer screening services, including conducting screening in more convenient locations; streamlining appointments to reduce the number of trips that people need to make to different healthcare settings; and increasing flexibility in relation to booking/rearranging screening appointments. Many participants felt that combining cancer screening and routine diabetes appointments would be helpful, though others suggested this might be ‘too much’ on 1 day.

From my house to the hospital, it's like £20 each way […] and I had seven appointments last week. P25 (aged 50; living with T2DM for 1–5 years; cervical screening = always; breast and bowel screening = not previously invited)

Especially in the early days when I was first diagnosed with my diabetes, I had quite a few appointments for different things. Um, if you then had a cancer screening appointment as well […] you might think […] I've had so much time off [work], I can't possibly ask for more time off for this. P14 (aged 53; living with T2DM for under a year; cervical screening = sometimes; breast screening = never; bowel screening = not previously invited)

All these different screenings, whether they be diabetic or otherwise, um, I mean they don't always coincide. […] It'd be nice to just go and get them all done at the same time. Um, but then they're not always in the same place. P11 (aged 60; living with T2DM for 1–5 years; cervical, breast and bowel screening = always)

#### Reduced psychological capacities to participate in cancer screening

Having a high burden of illness and its treatment often appeared to be mentally draining for people with T2DM. Participants frequently spoke of feeling overwhelmed and experiencing feelings of anxiety and low mood, related to living with and managing diabetes and comorbidities, leaving less available psychological ‘bandwidth’ for participating in cancer screening. For example, some participants experienced difficulties remembering to participate in screening, whilst others could feel overfaced by having to attend yet ‘another’ appointment. Concerns were raised that some people with T2DM may ignore their diabetes and other aspects of their health (e.g. cancer screening) due to feelings of overwhelm or low mood. To buffer feelings of lower psychological resource, participants sometimes needed other people (e.g. family members) to help them arrange or remember appointments, or to accompany them to cancer screening tests (e.g. for emotional support).

It [managing diabetes] still does get overwhelming sometimes and you just think, ‘oh my God, I'm having to do this for the rest of my life’. When, you know, you have good days and bad days. You know, and sometimes you just feel, ‘oh God, why am I bothering?’ P10 (aged 59; living with T2DM for 1–5 years; cervical screening = sometimes; breast screening = always; bowel screening = not previously invited)

The majority of people that I've spoken to, they just deny it [diabetes]. Or they think, ‘I can't do anything about it’. And, and screening then is another thing, ‘oh what are they gonna tell me?’ P6 (aged 60; living with T2DM for under a year; cervical, breast and bowel screening = always)

When I was diabetic and very overweight and very depressed, all those things, it was much, much harder for me to take part [in cancer screening] […] it is hard to get motivated or even, the sort of feel you're worth it. P8 (aged 66; living with T2DM for 5–10 years; cervical, breast and bowel screening = always)

### Diabetes complicates participation in cancer screening tests

#### Diabetes‐related physical difficulties during the cancer screening process

Participants sometimes faced additional physical difficulties related to their diabetes, its treatment, complications or common comorbid conditions, which complicated undergoing cancer screening tests. For example, people with T2DM frequently experienced loose and unpredictable bowel movements, which they typically attributed to the diabetes medication metformin. In contrast, a couple of participants experienced constipation. Such problems could make collecting a solid sample for bowel screening more difficult or unpleasant, with some individuals needing to ‘choose their day’ to complete the test. Some diabetes complications, such as skin infections, frozen shoulder and vaginal thrush, could make cancer screening tests more difficult, unpleasant or even impossible, whilst being overweight could result in increased pain and difficulties with positioning during tests. Women with T2DM occasionally raised concerns that people who are overweight may receive less accurate cancer screening and encounter accessibility difficulties, as screening facilities are not designed to accommodate them.

When you're taking medication like metformin, you don't have firm bowel movements […] it's um, quite the opposite, and it can come on you really suddenly. So it'd be hard to plan that, in a way that would actually allow you to collect it [a faeces sample]. If it, when it's well formed, it's much, much easier. P8 (aged 66; living with T2DM for 5–10 years; cervical, breast and bowel screening = always)

They couldn't do the cervical screening because there was too much of that medication still there […] I think it may have been a thrush treatment. P14 (aged 53; living with T2DM for less than a year; cervical screening = sometimes; breast screening = never; bowel screening = not previously invited)

A lot of them [healthcare professionals] don't understand the difficulties of a larger‐size lady […] having the over‐, aprons, overhang, maybe the skin infections […] it can be quite painful […] a lot of larger ladies can't lay flat on their back, you know, they need propping up a little bit. P19 (aged 59; living with T2DM for 1–5 years; cervical and breast screening = always; bowel screening = not previously invited)

#### Having to consider glycaemic control during cancer screening tests

People with T2DM, particularly those taking diabetes medications, sometimes had to make additional practical considerations related to diabetes management when attending cancer screening appointments. A few women with T2DM highlighted that they could, on occasion, experience blood sugar level changes during screening (e.g. due to stress or long waiting times). Needing to be prepared for managing blood sugar fluctuations (e.g. by taking diabetes medications or snacks, or avoiding driving, to appointments) was a source of additional stress and hassle for some individuals. Consequently, participants occasionally noted that cancer screening facilities might consider storing refreshments to provide to people with diabetes if they become hypoglycaemic during their appointment.

It is worrying, not knowing, oh, am I going to need to inject my insulin? […] It's constantly having to think about things ahead. P10 (aged 59; living with T2DM for 1–5 years; cervical screening = sometimes; breast screening = always; bowel screening = not previously invited)

If I got anxious having the mammogram and my blood sugars dropped, then I wouldn't be able to drive myself back. P27 (aged 70; living with T2DM for over 10 years; cervical, breast and bowel screening = always)

If you're waiting a long time, it, it's, it's a bit frightening sometimes, because you've got, if you've got no access to food or drink […] if you're going to an appointment like that, you would either take something with you, or you would make sure you eat before you go, which I do. And generally, you're not waiting for that long [at cancer screening appointments]. P36 (aged 65; living with T2DM for over 10 years; cervical and breast screening = always; bowel screening = sometimes)

#### Stigma and embarrassment related to diabetes

Many participants believed people hold stigmatizing attitudes towards those with T2DM (e.g. T2DM is a self‐inflicted illness caused by being overweight). For some participants, this appeared to contribute to felt stigma and embarrassment, and sometimes related feelings of low self‐esteem and self‐worth, which could discourage participation in cancer screening tests. For example, some participants spoke of experiencing embarrassment when undressing in front of healthcare professionals, because of their weight or diabetes complications. Several participants felt they sometimes received stigmatizing attitudes or poor care from professionals, and it was noted that people with T2DM may come to avoid healthcare settings, though some participants stated that perceived stigma or feeling embarrassment would not prevent them from undergoing screening. A few women indicated that they would like healthcare professionals to take time to help them feel more at ease and less self‐conscious during screening, whilst others wanted reassurance from clinicians that they are not being negatively judged for having T2DM or being overweight.

I have had a taste of it myself, that you're blamed for having type 2 diabetes. There's an attitude of blame, um, and humiliation, if you've got weight problems as well as having diabetes, as if you, you're responsible, you've caused it yourself. P12 (aged 67; living with T2DM for over 10 years; cervical, breast and bowel screening = always)

The other thing is being made to feel uncomfortable, because you're [laughs] bigger, you know, especially with the […] mammography. […] They say things like, ‘oh we can't fit you all on one plate’ […] it's hard, if you've been through a painful experience like that, it must be hard to go back. P8 (aged 66; living with T2DM for 5–10 years; cervical, breast and bowel screening = always)

Why do you have to strip off [for breast screening]? You could just lift your top up at the side […] and then you feel a bit more covered up, don't you? If you're overweight. P30 (aged 65; living with T2DM for over 10 years; cervical and breast screening = always; bowel screening = sometimes)

### Low promotion of cancer risk and screening within diabetes care

#### Low awareness of the increased risk of cancer associated with T2DM

Most women with T2DM appeared to be unaware of the increased risk of developing bowel and breast cancers associated with T2DM. A few women suspected that people with diabetes may be more likely to develop illnesses such as cancer, but were uncertain about this, or believed that any increased risk would only apply to people with poorly controlled diabetes. Only a couple of participants were confident that people with T2DM have an increased risk of developing cancer, though they did not appear to know specifically about the T2DM‐increased risk of bowel and breast cancers. A small number of participants perceived a lower personal risk of developing cancer due to lifestyle changes made following their diabetes diagnosis. In contrast, high awareness of other diabetes‐related health conditions (e.g. cardiovascular disease, eye and foot problems) was widely evident. Most participants did not perceive cancer screening to be more important for people with diabetes, though some did, usually due to other reasons than the T2DM‐increased cancer risk (e.g. anticipated burden of dealing with another diagnosis; feeling more vulnerable to illnesses in general).

I mean, we don't really get, we don't really get cancers, do we, from diabetes? It's more the, the, the strains on the heart, isn't it? P2 (aged 53; living with T2DM for 1–5 years; cervical and breast screening = always; bowel screening = not previously invited)

Apart from your eyes and your feet, I've not been told, you know, that you, that anything else can happen because of the diabetes. P10 (aged 59; living with T2DM for 1–5 years; cervical screening = sometimes; breast screening = always; bowel screening = not previously invited)

If you're more prone to picking up a certain, um, cancer or whatever [as someone with diabetes], then yes, it would be advisable to have that information, so that you can look at it and think, ooh yeah, I need to go [to screening]. P21 (aged 59; living with T2DM for less than a year; cervical and breast screening = always; bowel screening = not previously invited)

#### Limited cancer screening promotion and support from diabetes care providers

Participants indicated that their diabetes care providers frequently informed them about diabetes‐related health conditions (e.g. diabetic retinopathy) and encouraged participation in related self‐care activities (e.g. diabetic eye screening). In contrast, the T2DM‐increased risk of cancer and the importance of cancer screening participation were rarely covered during routine diabetes appointments, though one or two participants noted that occasions of encouragement or support to participate in cancer screening during diabetes‐related appointments had facilitated their participation. Some women expressed a desire to be informed about the T2DM‐increased risk of cancer, and/or indicated that they would find cancer screening‐related encouragement from their diabetes care provider beneficial. However, other women expressed reservations about discussing cancer risk and screening with diabetes providers (e.g. concerns that knowledge of the T2DM‐increased cancer risk may cause some anxiety or diabetes professionals lack cancer‐related knowledge).

I went [to cervical screening] a few years, probably two or three years ago, I was actually persuaded by this diabetes nurse. Because she asked me about it […] when I was there [at their diabetes check‐up]. P7 (aged 56; living with T2DM for 1–5 years; cervical and breast screening = sometimes; bowel screening = always)

At your, your annual [diabetes] review, they could remind you to make sure you go for your smear and the mammogram […] I do have friends who haven't taken up on these offers, so maybe a little drip feed from the diabetes team would help. P23 (aged 55; living with T2DM for less than a year; cervical and breast screening = always; bowel screening = not previously invited)

I'd just rather discuss my diabetes [at routine diabetes appointment]. […] I think it gets too confusing if they start talking about anything else, because then I start panicking, thinking, oh my God, am I gonna get this? Or is this something that's the next thing up? P36 (aged 65; living with T2DM for over 10 years; cervical and breast screening = always; bowel screening = sometimes)

## DISCUSSION

To our knowledge, this is the first in‐depth qualitative interview study to explore diabetes‐related barriers to cervical, breast and bowel cancer screening, and potential ways to address these, among women with T2DM, living in England (i.e. a country offering organized screening programmes as part of universal healthcare). Findings indicate that having diabetes can heighten commonly experienced barriers to cancer screening, and simultaneously pose additional diabetes‐specific barriers, despite high self‐reported cancer screening uptake among the study sample. Many women with T2DM experienced an accumulated high burden of illness and its treatment, due to diabetes and comorbidities, which consumed their personal resources, reducing available capacities for participation in cancer screening. Additionally, having diabetes could complicate taking part in screening tests for some women with T2DM, by presenting additional physical, practical and psychological barriers. Findings also indicated low awareness of the T2DM‐increased risk of bowel and breast cancers, and limited promotion of cancer screening within diabetes care.

Many participants experienced a high burden of illness and its treatment, which drew upon their physical, practical and psychological resources, reducing their available capacities for participation in cancer screening. This served to heighten commonly experienced barriers to cancer screening. Previous work, conducted in a range of countries, has similarly demonstrated a high symptom (Cannon et al., 2018; Sudore et al., 2012) and illness work (Buffel du Vaure et al., 2016; Herzig et al., 2019; Jowsey et al., 2012) burden among people with diabetes. Moreover, most people with T2DM have comorbidities (Iglay et al., 2016). Consistent with the current study, the presence of comorbidities has been shown to exacerbate the symptom (Cannon et al., 2018) and illness work (Buffel du Vaure et al., 2016) burden experienced by people with diabetes. Previous qualitative studies have identified having a high burden of illness and its treatment as a barrier to cancer screening among individuals with chronic illnesses (e.g. HIV; chronic kidney disease) (Bukirwa et al., 2015; James et al., 2017; Tarasenko & Schoenberg, 2011); this barrier was also identified by a study which included people with diabetes alongside participants with other health conditions (Shaw et al., 2012). To our knowledge, the current study is the first to highlight how the accumulated burden of diabetes, and its treatment and comorbidities, can make cancer screening participation more difficult for women with T2DM (e.g. by heightening travel‐related or scheduling barriers). Participants in this study described receiving cancer screening‐related practical and emotional support from other people (e.g. family members), which helped to buffer their reduced capacities. In contrast, although women with diabetes in Marshall's (2014) study did receive support from their family and friends, this support typically focused on their diabetes, rather than cancer screening.

Diabetes could also uniquely complicate taking part in cancer screening tests. Some participants experienced physical difficulties during screening, due to diabetes, its treatment, complications or common comorbid conditions. For example, loose, unpredictable or infrequent bowel movements, which can occur due to diabetes complications or medications (Piper & Saad, 2017), could make bowel screening more difficult or unpleasant. This novel finding is important given that many patients who take metformin, a first‐line T2DM medication recommended globally, experience gastrointestinal side effects (Nabrdalik et al., 2022). Previous studies, conducted in the USA, have also identified overweight‐related, physical barriers to cervical and breast screening (e.g. unsuitable medical equipment) (Amy et al., 2006; Friedman et al., 2012). Some women with T2DM in the current study spoke of the additional hassle of needing to consider their glycaemic control during cancer screening tests. Similarly, a participant with diabetes in a previous qualitative study highlighted how long waiting times at medical appointments can negatively impact glycaemic control (Hipwell et al., 2014). Moreover, a woman with diabetes in Marshall's (2014) study expressed concern that cancer screening‐related stress could affect their blood sugar levels. Participants also described experiencing diabetes and overweight‐related stigma, including within healthcare settings, which aligns with findings from USA‐based quantitative studies (Himmelstein & Puhl, 2021; Puhl et al., 2020). Perceived overweight‐related stigma and embarrassment have previously been identified as barriers to cervical and breast screening participation among women with obesity in the USA (Amy et al., 2006; Friedman et al., 2012). HPV self‐collection may provide a less embarrassing and more convenient alternative to in‐clinic cervical screening for women with T2DM (Mkuu et al., 2024).

Participants often seemed to lack awareness of the T2DM‐increased risk of breast and bowel cancer, despite being aware of other diabetes‐related health conditions; this finding is concurrent with results from a large British survey study by Ashley et al. (2023), and Marshall's (2014) USA‐based qualitative study. The current study provides some support for the suggestion by Ashley et al. (2023) that this lack of awareness may be partly due to a lack of information provision from diabetes care providers and organizations. Perceived cancer risk is positively associated with screening participation (Young & Robb, 2021). Qualitative studies have highlighted having a lack of awareness of being at an increased risk of developing cancer as a barrier to cancer screening participation and/or informed decision‐making among individuals with HIV (Williams et al., 2015) and kidney transplant recipients (Williams et al., 2012), whilst awareness of the HIV‐increased risk of cancer has been found to facilitate screening participation (Fletcher et al., 2014; Williams et al., 2015). Novel to this study, participants occasionally highlighted how opportunistic cancer screening‐related support or encouragement received during diabetes‐related medical appointments was facilitative of cancer screening participation. It is possible that integrating promotion of T2DM‐increased cancer risk and cancer screening participation into diabetes care, consistent with calls by Ashley et al. (2023), would benefit cancer screening decision‐making and uptake among people with T2DM. Both Marshall (2014) and Mkuu et al. (2024) identified healthcare professional endorsement as a facilitator to cancer screening among women with diabetes, thus providing support for this suggestion. However, it is important to note that these previous studies were conducted in a country offering opportunistic screening, which may have heightened individuals' perceived importance of receiving provider recommendations (Wardle et al., 2015).

### Strengths and limitations

Strengths of this study include the recruitment of a sample with some diversity, on several sociodemographic and diabetes‐related characteristics (i.e. age; highest level of education; relationship status; employment status; length of time living with T2DM; type of diabetes management), via multiple channels (i.e. NHS diabetic eye screening clinics across England; diabetes charities; social media). Interviews were generally in‐depth and informed by a comprehensive topic guide, which drew upon previous research and the TDF (Cane et al., 2012; Michie et al., 2005). A limitation of this study is that participants generally self‐reported high engagement with cancer screening, whereas ideally our sample would have included more women with lower screening uptake. Research has suggested, however, that people may over‐report their participation in cancer screening (Howard et al., 2009; Lo et al., 2015; Rauscher et al., 2008). For example, in an English survey study, consistent uptake of bowel cancer screening was substantially higher as self‐reported than according to medical records (82% vs. 66%) (Lo et al., 2015). Also, all participants who self‐reported their ethnicity described themselves as “White”, which is a limitation particularly given that, in the UK, cancer screening uptake is lower, and T2DM prevalence is higher, among non‐white ethnic groups (Goff, 2019; Young & Robb, 2021). Previous qualitative studies have similarly struggled to recruit people who have no or low participation in cancer screening and people from minority ethnic groups (McWilliams et al., 2021; Nemec et al., 2022; O'Donnell et al., 2023; Travis et al., 2024). Future qualitative work should seek to address these limitations by incorporating strategies to help improve the recruitment of underserved groups (e.g. snowball sampling; establishing relationships with community leaders prior to participant recruitment; multilingual recruitment materials) (Ellard‐Gray et al., 2015; Langer et al., 2021; Rockliffe et al., 2018), which funding constraints prevented in the current study. In addition to research interviews, future work might also seek to more successfully engage underrepresented groups in the context of obtaining evaluative feedback on experienced real world clinical practice changes, such as incorporation of cancer screening promotion into routine diabetes health checks, discussed below.

### Implications for clinical practice

Based on the findings of this study, we have developed practice recommendations which are detailed in Table 2. To our knowledge, this is the first comprehensive list of recommendations to improve cancer screening informed decision‐making and participation among people with T2DM. Many of the recommendations centre around potential adjustments to cancer screening services to help mitigate diabetes‐related barriers to engagement, whilst others relate to integrating promotion of cancer risk and screening into diabetes care. Whilst some recommendations may require significant resources to implement (e.g. patient transport support to attend screening appointments), others may be comparatively easier to implement (e.g. promoting cancer screening participation at a routine diabetes review). There is growing recognition of the potential usefulness of integrating cancer screening promotion in routine chronic disease reviews (e.g. Suh & Kim, 2019). In the UK, recent government guidance to local Cancer Alliances around strategies to reduce inequalities in early cancer diagnosis includes incorporating checks or prompts on cancer screening into chronic disease reviews. Following the findings of this study and other research by our team (e.g. Ashley et al., 2023), a clinician‐led regional pilot is underway exploring the acceptability and feasibility, and cancer screening uptake outcomes, of incorporating into annual diabetes reviews clinician‐provided education on T2DM‐increased cancer risk, personalized discussion around experienced barriers to cancer screening, and encouragement and support to take‐up any current screening invitations.

**TABLE 2:** Recommendations to improve cancer screening informed decision‐making and participation among people with type 2 diabetes (T2DM).

Recommendations	
**Reducing the burden of attending cancer screening**	
1.	Ensure flexibility for booking and rearranging cancer screening appointments (e.g. *offer wider choice of appointment times; provide clear information and reminders about how to rearrange appointments*)
2.	Streamline appointments where possible and desired (e.g. *cervical screening at the same visit as a diabetes review*)
3.	Mitigate challenges around travel to cancer screening appointments (e.g. *offer at home self‐sampling cervical screening; assist with accessing non‐emergency patient transport services where eligible*)
**Improving the accessibility of cancer screening for people with diabetes**	
4.	Increase the physical accessibility of cancer screening facilities (e.g. *disabled parking, wheelchair access*)
5.	Provide continuing education to cancer screening staff around effectively supporting people with diabetes and overweight or obesity during screening tests (e.g. *comfortable positioning during cervical screening; enabling patients to remain as covered up as possible during mammograms, such as by providing a hospital gown; trust‐building interactions sensitive to potential patient felt embarrassment and stigma*)
6.	Store refreshments at cancer screening facilities to provide to people with diabetes if they become hypoglycaemic during their appointment
**Integrating promotion of cancer risk and screening into diabetes care**	
7.	Include within information about diabetes‐related health conditions the T2DM‐increased risk of bowel and breast cancers and related benefit of cancer screening participation
8.	Promote and support cancer screening uptake at routine diabetes review appointments (e.g. *help address any diabetes‐related barriers to screening, such as challenges bowel testing due to loose stools*)
9.	Provide tailored cancer screening non‐attendance follow‐up reminders (e.g. *highlighting T2DM‐increased cancer risk, sent from the practice diabetes nurse*)

## CONCLUSION

This in‐depth qualitative interview study provides the first detailed exploration of barriers to cervical, breast and bowel cancer screening among women with T2DM, living in England. Findings indicate that having diabetes can heighten commonly experienced barriers to cancer screening (e.g. travel‐related, logistical and scheduling barriers) and pose additional diabetes‐specific barriers to screening (e.g. diabetes‐related stigma and embarrassment). The accumulated high burden of illness and its treatment, due to diabetes and comorbidities, consumes personal resources, reducing available capacities (e.g. energy, time, money) to participate in cancer screening. Additionally, having diabetes can complicate taking part in screening tests (e.g. diabetes medication side effects include loose stools, which make bowel cancer tests more difficult to complete; T2DM‐related stigma and embarrassment can discourage attendance at mammograms). There is also underappreciation of the T2DM‐increased risk of breast and bowel cancer, and limited cancer screening promotion within diabetes care. We present the first comprehensive list of recommendations to improve cancer screening informed decision‐making and participation among people with T2DM.

## AUTHOR CONTRIBUTIONS

**Rebecca Spencer:** Conceptualization; investigation; formal analysis; writing – original draft; writing – review and editing. **Georgina Jones:** Supervision; formal analysis; writing – review and editing. **Siobhán McHugh:** Supervision; formal analysis; writing – review and editing. **Ramzi A. Ajjan:** Funding acquisition; supervision; writing – review and editing. **Rebecca J. Birch:** Supervision; writing – review and editing. **Laura Ashley:** Funding acquisition; conceptualization; supervision; formal analysis; writing – review and editing.

## Data Availability

The terms of the participants' consent do not allow raw data to be made publicly available.
